# Artemisinin-based combination therapy during pregnancy: outcome of pregnancy and infant mortality: a cohort study

**DOI:** 10.1186/s12936-019-2737-7

**Published:** 2019-03-28

**Authors:** Michael Nambozi, Halidou Tinto, Victor Mwapasa, Harry Tagbor, Jean-Bertin Bukasa Kabuya, Sebastian Hachizovu, Maminata Traoré, Innocent Valea, Marc Christian Tahita, Gifty Ampofo, Jozefien Buyze, Raffaella Ravinetto, Diana Arango, Kamala Thriemer, Modest Mulenga, Jean-Pierre van Geertruyden, Umberto D’Alessandro

**Affiliations:** 1 Tropical Diseases Research Centre, Ndola, Zambia; 2 Institut de Recherche en Sciences de la Santé-Clinical Research Unit of Nanoro (IRSS-CRUN), Nanoro, Burkina Faso; 3 College of Medicine, Blantyre, Malawi; 4 University of Health and Allied Science, Ho, Ghana; 5 Department of Clinical Sciences, Institute of Tropical Medicine, Antwerp, Belgium; 6 Department of Public Health, Institute of Tropical Medicine, Antwerp, Belgium; 7 Menzies School of Health Research, Darwin, Australia; 8 Global Health Institute, University of Antwerp, Antwerp, Belgium; 9 Medical Research Council Unit, The Gambia at the London School of Hygiene and Tropical Medicine, Fajara, Gambia

## Abstract

**Background:**

The World Health Organization (WHO) recommendation of treating uncomplicated malaria during the second and third trimester of pregnancy with an artemisinin-based combination therapy (ACT) has already been implemented by all sub-Saharan African countries. However, there is limited knowledge on the effect of ACT on pregnancy outcomes, and on newborn and infant’s health.

**Methods:**

Pregnant women with malaria in four countries (Burkina Faso, Ghana, Malawi and Zambia) were treated with either artemether–lumefantrine (AL), amodiaquine–artesunate (ASAQ), mefloquine-artesunate (MQAS), or dihydroartemisinin–piperaquine (DHA–PQ); 3127 live new-borns (822 in the AL, 775 in the ASAQ, 765 in the MQAS and 765 in the DHAPQ arms) were followed-up until their first birthday.

**Results:**

Prevalence of placental malaria and low birth weight were 28.0% (738/2646) and 16.0% (480/2999), respectively, with no significant differences between treatment arms. No differences in congenital malformations (p = 0.35), perinatal mortality (p = 0.77), neonatal mortality (p = 0.21), and infant mortality (p = 0.96) were found.

**Conclusions:**

Outcome of pregnancy and infant survival were similar between treatment arms indicating that any of the four artemisinin-based combinations could be safely used during the second and third trimester of pregnancy without any adverse effect on the baby. Nevertheless, smaller safety differences between artemisinin-based combinations cannot be excluded; country-wide post-marketing surveillance would be very helpful to confirm such findings.

*Trial registration* ClinicalTrials.gov, NCT00852423, Registered on 27 February 2009, https://clinicaltrials.gov/ct2/show/NCT00852423

## Background

Pregnant women are more vulnerable to malaria than non-pregnant women because of altered immunity. Each year, about 85 million pregnant women in sub-Saharan Africa (SSA) are at risk of *Plasmodium falciparum* infection [1, 2]. *Plasmodium falciparum* malaria in pregnancy (either asymptomatic or symptomatic) can result in serious adverse outcomes for both the mother and the infant; maternal anaemia and placental malaria may result in low birth weight (LBW), miscarriage, stillbirth and infant death [3–6]. Effective and safe anti-malarial treatments decrease the occurrence of such adverse outcomes. The World Health Organization (WHO) recommends the use of artemisinin-based combination therapy (ACT) for the treatment of *P. falciparum* uncomplicated malaria during the second and third trimester of pregnancy [7]. All sub-Saharan African countries have already adopted and implemented such recommendation. However, there is limited knowledge on the effect of ACT use on pregnancy outcomes and infant’s safety. Most of the available information originates from South-East Asia while there is little information from sub-Saharan Africa [8–11]. None of the studies in South-East Asia found evidence of fetal or maternal toxicity associated with artemisinin derivatives or ACT, with rate of abortion, congenital abnormalities, and stillbirths within normal community ranges [9–11].

The results of the safety and efficacy of four artemisinin-based combinations, namely artemether-lumefantrine (AL), amodiaquine-artesunate (ASAQ), mefloquine-artesunate (MQAS), dihydroartemisinin–piperaquine (DHA–PQ), in African pregnant women with malaria were recently reported [12, 13]. AL had the best tolerability profile, acceptable cure rates, but the shortest post-treatment prophylaxis, while DHA–PQ seemed the most suitable treatment in terms of safety and efficacy, including its longer post-treatment prophylaxis [12, 13]. Here, the effect of the four artemisinin-based combinations on pregnancy outcome, and on neonatal and infant morbidity and mortality are reported.

## Methods

The study was an open label, randomized controlled clinical trial comparing the efficacy and safety of four artemisinin-based combinations in pregnant women with *P. falciparum* uncomplicated malaria in the second and third trimester of pregnancy. The trial protocol has been reported in detail elsewhere (NCT00852423) [14]. Briefly, the trial was carried out between June 2010 and April 2015 at seven sites across four countries, namely Burkina Faso, Ghana, Malawi and Zambia. Pregnant women in their second or third trimester were enrolled and randomized to one of four treatment arms (AL, ASAQ, MQAS and DHAPQ). Participants were followed up until day 63 and then were seen at delivery.

## Infant follow-up

Mothers were asked to attend study health facilities with their babies at 4–6 weeks post-delivery and/or at the time of due vaccinations, 6 months later and at around the baby’s first birthday. Infants were examined by a study nurse or doctor and their medical conditions, if any, managed as appropriate, including hospital admissions. Only congenital anomalies or birth defects and deaths were reported as serious adverse events (SAEs), and relevant information was collected by interviewing the mother and/or from hospital records. SAEs were codified using Medical Dictionary for Regulatory Activities (MeDRA) preferred term.

Perinatal mortality rate was defined as the number of perinatal deaths (stillbirths and early neonatal deaths) per 1000 total births [15]. Stillbirth was defined as a baby born dead after 24 weeks of gestation while miscarriage was defined as loss of the fetus at 24 weeks of gestation or earlier. Neonatal mortality rate was defined as the number of neonatal deaths (death during the first 28 days of life) per 1000 live births (first week of life: early neonatal death; 8–28 days of life: late neonatal death) [16]. Infant mortality rate was defined as the number of infant deaths (< 1 year of age) per 1000 live births. Placental malaria was classified as acute and chronic infection (presence of parasites with or without malaria pigment), past infection (presence of malaria pigment) and no infection (no parasites or malaria pigment).

The trial was approved by the ethics committee of the Antwerp University Hospital, Belgium, the relevant national or local ethics committees, and the national drug regulatory authorities of the four African participating countries.

## Statistical analysis

The data analysis was based on an intention-to-treat (ITT) analysis, i.e. all infants born to mothers included in the study and randomized to one of the four treatments were included, regardless of the number of doses taken. Nevertheless, denominator of percentages varied according to the availability of information; this explains the variation of denominator by variable. Twins were excluded from the analysis as mortality is higher in this group [17]. Descriptive statistics were used to summarise socio-demographic and clinical characteristics of the mothers and infants. The difference between the treatment arms was calculated using logistic regression with fixed effects for treatment and country. The same approach was applied for the incidence of fever and other symptoms such as cough, diarrhoea, and difficulty in feeding at 4–6 weeks of life per 1000 live births. Continuous variables were compared between treatment groups using ordinary least square regression adjusted for country.

Incidence of SAEs in infants between treatment groups was compared by using a logistic regression model with fixed effects for treatment and country, to correct for possible imbalances in the reporting between countries.

Population-attributable fraction was used to assess the effect of placental malaria on LBW. The prevalence ratio (Pr) was set as the proportion of infected placentas (acute and chronic infections) with LBW divided by the proportion of uninfected placenta (past infections and uninfected) with LBW. The infected attributable fraction was calculated as the percentage of infected placenta with LBW that was due to malaria [(Pr − 1)/Pr] while the population-attributable fraction was the percentage of LBW cases that were due to malaria infection [18].

Infant mortality and hospital admissions rates were calculated as number of subjects with the event over the time at risk. The rate was compared between treatment arms using Poisson regression or negative binomial regression model [19] adjusted for covariates: country, treatment, maternal age, delivery (gestational age, Hb, parasite density, baby Hb), birth weight, birth asphyxia, mode of delivery, congenital malformation, gravidity, placental malaria, place of delivery, ITN use by mother or baby, intermittent preventive treatment (IPTp) use, hospital admission, malaria infection in new-born. In the univariate analysis, covariates were selected for inclusion in multivariable model if the p-value was ≤ 0.25. In the final model covariates with a p-value of > 0.2 were dropped step by step. Stata v14 (Stata Corp, USA) was used for all statistical analyses. The significant level was set at p ≤ 0.05.

## Results

A total 3258 out of the 3428 mothers enrolled had a recorded delivery outcome. Maternal characteristics such as gestational age (estimated by the total Ballard score), and maternal malaria infection status at delivery were not significantly different between treatment groups (Table 1). Fifty-two mothers delivered twins and were excluded from analysis. Out of the total recorded delivery outcomes, 3127 mothers (excluding mothers with twins) delivered live babies: 822 in the AL, 775 in the ASAQ, 765 in the MQAS and 765 in the DHAPQ arms. There was no difference in the proportion of live births, adjusted for country, between treatment groups (p = 0.85) (Table 2). There were 13 (0.39%) miscarriages and 78 (2.36%) stillbirths [12]. Among all live births, 118/3127 (3.8%) were lost during the 1-year follow-up while 6 mothers (0.2%) withdrew their consent.

**Table 1:** Gestational age and maternal malaria infection status at delivery

	AL		ASAQ		MQAS		DHAPQ		p-value^a^
	*N*		*N*		*N*		*N*		
Maternal age median (IQR)	881	21 (18–26)	842^c^	22 (19–27)	850^c^	22 (19–27)	855	20 (18–25)	0.67
Gestational age (week) median (IQR)	804	38 (36–38)	729	38 (38–40)	733	38 (36–40)	709	38 (36–38)	0.30
Gravidity n (%)	880		842		850		855		0.55
1st pregnancy		319 (36.3)		315 (37.4)		278 (32.7)		343 (40.1)	
2nd pregnancy		204 (23.2)		187 (22.2)		201 (23.7)		216 (25.3)	
3rd or more		357 (40.6)		340 (40.4)		371 (43.7)		296 (34.6)	
Malaria prevalence (peripheral blood) n (%)	829	120 (14.5)	756	95 (12.6)	752	123 (16.4)	748	75 (10.0)	0.21
Parasite density; median (IQR)	120	1560 (440–6804)	95	1800 (320–8220)	123	1729 (561–9000)	75	2320 (480–8960)	0.09
% ≤ 2000/µL (n)		55.0 (66)		53.7 (51)		52.0 (64)		46.7 (35)	
% > 2000/µL (n)		45.0 (54)		46.3 (44)		48.0 (59)		53.3 (40)	
Gametocyte carriage n (%)	829	3 (0.4)	756	4 (0.5)	752	4 (0.5)	748	2 (0.3)	0.89
Maternal Hb median (IQR)	828	11.2 (10.1–12.2)	757	11.5 (10.4–12.4)	752	11.3 (10.3–12.3)	749	11.2 (10.3–12.2)	0.30
Placenta Malaria n (%)^b^	711		655		674		664		
Acute infection		7 (1.0)		14 (2.1)		13 (1.9)		11 (1.7)	
Chronic infection		191 (26.9)		168 (25.6)		177 (26.3)		171 (25.8)	
Past infection		444 (62.4)		382 (58.3)		398 (59.1)		409 (61.6)	
No infection		69 (9.7)		91 (13.9)		86 (12.8)		73 (11.0)	

^a^Adjusted by country

^b^The proportion of current (acute and chronic) and past infection vs. no infection, adjusted for country, p = 0.47. Proportion of acute and chronic infection vs. past or no infection, adjusted for country p = 0.52

^c^One woman randomized to the ASAQ group was treated with MQAS; in the safety analysis this woman was included in the MQAS group

**Table 2:** Baseline characteristics of study infants at delivery (%)

	AL		ASAQ		MQAS		DHAPQ		p-value^a^
	*N*	Value	*N*	Value	*N*	Value	*N*	Value	
Live births by country									0.85
Burkina Faso	280	278 (99.3)	279	278 (99.6)	275	268 (97.5)			
Ghana			245	235 (95.9)	241	229 (95.0)	252	243 (96.4)	
Malawi	276	269 (97.5)	269	262 (97.4)			270	268 (99.3)	
Zambia	282	275 (97.5)			273	268 (98.2)	264	254 (96.2)	
Birth asphyxia	706	19 (2.7)	635	42 (6.6)	658	44 (6.7)	648	39 (6.0)	0.91
Congenital abnormality^b^	815	17 (2.1)	757	12 (1.6)	751	12 (1.6)	742	6 (0.8)	0.35
Prematurity^c^	838	78 (9.3)	793	25 (3.2)	789	57 (7.2)	786	70 (8.9)	0.65
Congenital malaria^d^	808	7 (0.9)	727	2 (0.3)	719	1 (0.1)	711	1 (0.1)	0.15
Anaemia at birth^d^	793	12 (1.5)	720	7 (1.0)	713	10 (1.4)	696	10 (1.4)	0.36
Birth weight mean (SD)	804	2856 (452)	742	2873 (463)	733	2860 (460)	720	2889 (463)	0.56
LBW < 2500 g	804	138 (17.2)	742	118 (15.9)	733	119 (16.2)	720	105 (14.6)	0.54

^a^Adjusted by country

^b^Reported as SAE at delivery

^c^Calculated by Ballard score

^d^Cord blood at 14 g/dL cut off for congenital anaemia and cord blood for malaria

Prevalence of LBW was 16.0% (480/2999), and was significantly more frequent among women with acute and chronic placenta malaria (21.0%, 152/723) than in those with past or no infection (13.7%, 259/1892) (p < 0.01). Nevertheless, LBW (p = 0.54) and mean birth weight were similar between treatment arms (Table 2). Prevalence of both cord blood anaemia and congenital malaria infection (malaria parasites in cord blood) was low and not significantly different between treatment arms. Similarly, there were few congenital abnormalities identified at birth (1.4%, 44/3065), with no statistically significant difference between study arms (Table 2) [12].

The overall prevalence of acute and chronic placental malaria was 28.0% (738/2646). Attributable fraction analysis suggests placenta malaria (acute and chronic) was responsible for 35% of the LBW among women with placental malaria infection (infected attributable fraction). At the prevalence found in this trial, the percentage of all LBW babies due to malaria (population-attributable fraction) was estimated at 13% (Table 3).

**Table 3:** Risk of LBW associated with placental malaria

Placenta malaria prevalence	Prevalence of LBW		Prevalence ratio	Infected attributable fraction	Population-attributable fraction
	Placenta malaria	No Placenta malaria			
27.89 (738/2646)	21.0 (152/723)	13.7 (259/1892)	1.535	34.87	12.99

The prevalence ratio (Pr) is the proportion of infected women with LBW divided by the proportion of uninfected women with LBW. The infected attributable fraction is the percentage of infected women with LBW that is due to malaria [(Pr − 1)/pr]. The population-attributable fraction is the percentage of LBW cases that are due to malaria infection: [PM(Pr − 1)]/{1 + [PM(Pr − 1)]} where PM is the proportion with placental malaria infection

Besides congenital abnormalities (almost half of them (46%, 23/50) polydactyly), the most commonly reported SAEs among infants were infections and infestations (Table 4), the most frequent being pneumonia (26.1%, 12/46), neonatal sepsis (21.7%, 10/46), sepsis (17.4%, 8/46), and malaria (8.7%, 4/46), with no statistically significant difference between treatment arms.

**Table 4:** SAEs in infants by treatment arm

SAE by system organ class	AL (N = 822)		ASAQ (N = 775)		MQAS (N = 765)		DHAPQ (N = 765)	
	*%*	n	*%*	n	*%*	n	*%*	n
Congenital disorders	2.1	17	1.5	12	1.6	12	0.8	6
Infection and infestations	1.5	12	1.5	12	0.9	7	2.0	15
Respiratory disorders	1.0	8	0.8	6	0.9	7	1.0	8
General disorders	0.6	5	0.4	3	0.8	6	1.0	8
Gastrointestinal disorders	0.4	3	0.0	0	0.3	2	0.0	0
Perinatal complications	0.4	3	0.5	4	0.1	1	0.9	7
Blood disorders	0.2	2	0.0	0	0.1	1	0.1	1
Hepatobiliary disorders	0.1	1	0.0	0	0.0	0	0.0	0
Metabolism and nutrition disorders	0.1	1	0.1	1	0.0	0	0.3	2
Reproductive and breast disorders (e.g. labia enlarged)	0.1	1	0.0	0	0.0	0	0.0	0
Nervous system disorders	0.0	0	0.1	1	0.1	1	0.1	1

Reported hospital admissions (episodes per 1000 live birth/year) were similar between treatment arms: AL: 90, ASAQ: 104, MQAS: 56, DHAPQ: 85 (p = 0.91) (Table 5).

**Table 5:** Incidence of hospital admissions, infant mortality

Variable	N/PYAR	IR	IRR (95% CI)	p-value
Hospital admissions				
AL	67/748.6	0.09	Reference	
ASAQ	74/710.6	0.10	1.01 (0.71–1.43)	
MQAS	40/718.4	0.06	0.88 (0.57–1.35)	
DHAPQ	60/702.9	0.09	1.01 (0.70–1.47)	0.91
Infant mortality				
AL	29/748.6	0.04	Reference	
ASAQ	27/710.6	0.04	0.96 (0.44–2.09)	
MQAS	24/718.4	0.03	0.94 (0.47–1.86)	
DHAPQ	38/702.9	0.05	1.12 (0.57–2.20)	0.96

*IRR* incidence rate ratio

During the 1-year follow up, when adjusting for country and other potential risk factors, babies born at home (IRR 1.44, 95% CI 1.02–2.03) (p = 0.04) or with a congenital abnormality (IRR 5.12, 95% CI 2.09–12.51) were more likely to be admitted in hospital. Infants sleeping under bed nets (0.36, 95% CI 0.21–0.62) (p < 0.01) were less likely to be admitted in hospital than those who were not (Table 6).

**Table 6:** Risks (incidence rate ratio) of infant mortality and hospital admission in PREGACT study

	IRR	95% CI	p-value
Infant mortality			
AL	1	–	0.96
ASAQ	0.96	0.44–2.09	
MQAS	0.94	0.47–1.86	
DHAPQ	1.12	0.57–2.20	
Congenital anaemia	2.93	0.96–8.96	0.06
Low birth weight	1.78	1.03–3.06	0.04
Birth asphyxia	10.89	4.48–26.46	< 0.01
Congenital abnormality	25.47	10.46–62.02	< 0.01
Burkina Faso	1	–	< 0.01
Ghana	1.02	0.31–3.36	
Malawi	2.14	0.89–5.15	
Zambia	4.02	1.85–8.73	
Hospital admission			
AL	1	–	0.91
ASAQ	1.01	0.71–1.43	
MQAS	0.88	0.57–1.35	
DHAPQ	1.01	0.70–1.47	
Baby ITN use	0.36	0.21–0.62	< 0.01
Born at home	1.44	1.02–2.03	0.04
Congenital malaria	3.80	0.93–15.56	0.06
Congenital abnormality	5.12	2.09–12.51	< 0.01
Burkina Faso	1	–	< 0.01
Ghana	1.02	0.68–1.54	
Malawi	1.89	1.33–2.69	
Zambia	0.49	0.31–0.79	

*IRR* incidence rate ratio

There were 70 deaths within the first month of life, representing 59.3% (70/118) of all infant deaths, with no difference between study arms (55.2% in AL, 74.1% in ASAQ, 41.7% in MQAS and 63.2% in DHAPQ) (p = 0.47). The large majority of neonatal deaths (71.4%, 50/70) occurred in the first week of life, at the mean age of 1.3 (SD 1.9) days, with no statistically significant difference between treatment arms (AL: 2.1; ASAQ: 1.5; MQAS: 0.3; DHAPQ: 1.0) (p = 0.19). Neonatal mortality (early and late) rate was 22.4 deaths per 1000 live births (AL: 19.5; ASAQ: 25.8, MQAS: 13.1; and DHAPQ: 31.4) (p = 0.21); perinatal mortality rate was 21.9 deaths per 1000 total births (AL: 17.9; ASAQ: 17.7, MQAS: 28.0; and DHAPQ: 24.2) (p = 0.77), with no significant difference between the treatment groups (Table 7). The overall infant mortality rate was 41.0/1000 live births per year, and similar between treatment arms (AL: 38.7, ASAQ: 38.0, MQAS: 33.4 and DHAPQ: 54.1) (p = 0.96) (Table 5). Infant mortality was significantly higher in Zambia than in Burkina Faso but not in Ghana or Malawi (Table 6).

**Table 7:** Perinatal and neonatal mortality, and morbidity during the first month of life by treatment (%)

	AL		ASAQ		MQAS		DHAPQ		p-value^a^
	*N*	Value^b^	*N*	Value^b^	*N*	Value^b^	*N*	Value^b^	
Perinatal mortality	837	15 (17.9)	789	14 (17.7)	787	22 (28.0)	784	19 (24.2)	0.77
Neonatal mortality	822	16 (19.5)	775	20 (25.8)	765	10 (13.1)	765	24 (31.4)	0.21
Morbidity									
Fever (4–6 week)	764	37 (48.4)	718	34 (47.4)	715	42 (58.7)	680	23 (33.8)	0.82
Diarrhoea (4–6 week)	764	14 (18.3)	717	22 (30.7)	715	19 (26.6)	679	4 (5.9)	0.62
Cough (4–6 week)	764	34 (44.5)	717	33 (46.0)	715	56 (78.3)	679	52 (76.6)	0.02
Difficulty in feeding (4–6 week)	764	2 (2.6)	717	5 (7.0)	715	6 (8.4)	679	1 (1.5)	0.76
Jaundice (4–6 weeks)	764	0 (0.0)	717	1 (1.4)	714	2 (2.8)	679	1 (1.5)	0.90
Other symptoms (4–6 week)	764	33 (43.2)	717	44 (61.4)	715	51 (71.3)	679	16 (23.6)	0.33

^a^p-values are from logistic regression adjusted for country

^b^values are n (/1000 live births) except perinatal mortality which has n (/1000 total births)

When adjusting by country and other potential risk factors, LBW babies had an almost 2-fold higher risk of dying during the first year of life than other babies (IRR 1.78, 95% CI 1.03–3.06, p = 0.04). Babies born with birth asphyxia (IRR 10.89, 95% CI 4.48–26.46) (p < 0.01) and congenital abnormality (25.47, 95% CI 10.46–62.02) (p < 0.01) had significantly higher risk of dying than other infants (Table 6).

## Discussion

The PREGACT study aimed at evaluating the safety and efficacy of four artemisinin-based combinations when administered to pregnant women with malaria [12]. The follow-up included also the offspring’s first year of life to identify any potential problem the treatment may have. Results are reassuring as no significant differences between study arms in terms of reported illness, hospital admissions or infant mortality were found. It is extremely difficult to estimate cause-specific mortality as most infant deaths occurred outside a health facility [20–23]. Some of the most frequent SAEs reported in this study, namely neonatal sepsis, respiratory infections and malaria, are also among the most common causes of infant deaths in malaria-endemic African countries, where infant mortality rates are the highest in the world [19, 20], and were probably among the most frequent cause of infant death.

More than two-third of all neonatal deaths occurred in the first week of life. In this age group, malaria is usually an indirect cause of death as it causes LBW which in turn increases the risk of dying in the first year of life [20, 24]. This is confirmed by the significant association between LBW and placenta malaria (acute and chronic) and the substantially higher mortality in LBW babies. Few trials have evaluated the effect of chemoprevention during pregnancy on miscarriages, stillbirths, perinatal deaths, neonatal or infant deaths, and usually these studies are underpowered to detect clinically important differences [25] or do not follow infants until their first birthday [26, 27]. Such a difference may be found when comparing an intervention to a placebo arm, as in Mozambique where IPTp with sulfadoxine–pyrimethamine (SP) was compared to a placebo arm [28]. Indeed, in this trial significantly fewer neonatal deaths, particularly early infant deaths, occurred in the intervention than in placebo group despite no difference in prevalence of anaemia, LBW or placenta malaria (active and past infection) [28]. However, infant mortality did not differ between study arms although among the 58 infant deaths reported, more than half occurred in the placebo arm [20]. When all women included in a trial receive a malaria-preventive intervention, significant differences in neonatal and/or infant mortality are unlikely to be found unless one of the interventions has either a negative or positive effect on infant survival. For example, a trial comparing IPTp with either SP (IPTp-SP) or mefloquine (MQ) did not report any difference in neonatal and infant mortality between the two arms as both treatments are efficacious against malaria [29]. Therefore, the similar outcomes in the four arms of our trial are not surprising as all treatments were extremely efficacious against malaria [12]. A difference may have occurred if any of the treatments tested would have had an adverse effect on the fetus or infant survival or if the four tested artemisinin-based combinations were compared to a non-ACT. Indeed, in Uganda spontaneous abortions and neonatal deaths were less frequent among women treated with AL than in those treated with quinine although the difference was not statistically significant [30]. In Mali, the proportion of stillbirths (4.8%) and neonatal deaths (2.9%) after 2-dose IPTp-SP were similar to those observed in this trial (2.36% and 2.24%, respectively), confirming the safety of the ACT when given during the 2nd or 3rd trimester of pregnancy [31]. The early concerns about an increased risk of stillbirth associated with mefloquine [32] were not confirmed subsequently by other studies on mefloquine alone or combined with artesunate [33–35]; birth defects and fetal loss rates were similar to background rates [36] or to other antimalarial treatments [12, 37].

Placenta sequestration of malaria parasites can lead to inflammatory response, particularly in first-time mothers who often have high density infections, and also affect development of the fetal circulation. This decreases the supply of nutrients and oxygen to the fetus, resulting in intrauterine growth retardation and LBW, which is a risk factor for higher infant mortality [38]. More than a quarter of women had an active placenta infection, mainly chronic ones, i.e. with both parasites and malaria pigment, while evidence of past (non-active) infection was found in more than 60% of women. As the trial recruited pregnant women with malaria, such a high prevalence is understandable. In addition, women were actively followed up during the 63 days post-treatment, and passively until delivery. Most women were treated during the second trimester and the early third trimester of pregnancy, and the treatment by itself could not prevent women to be re-infected near delivery. Indeed, longitudinal genotyping of *P. falciparum* isolates during gestation in Cameroonian pregnant women showed that 77% of placental parasites were acquired from 30 weeks’ gestation onwards [38].

Fetal anaemia plays a role in neonatal survival [20, 39]. Cord blood anaemia was particularly low, just around 1% and similar in all treatment arms, although it increased the risk of infant death almost 3-fold, an association that was of borderline significance. Such low prevalence is surprising when considering that a recently published study on a trial assessing IPTp with either SP or MQ reported a prevalence of about 10%, with a definition of anaemia that was more conservative than in this study (Hb < 12.5 g/dl versus Hb < 14.0 g/dl) [29]. The reason for such difference is unclear.

The longer recall period between the post-partum visits, at 4–6 weeks, and then at 6 and 12 months could have affected the accuracy of information on morbidity. Nevertheless, it is unlikely the study team missed any infant death. Moreover, such recall bias would have equally affected the 4 study arms.

## Conclusions

In summary, there were no major differences between treatment arms in terms of perinatal, neonatal and infant mortality, nor on the overall occurrence of SAEs in babies, indicating that any of the tested artemisinin-based combinations can be used for the treatment of malaria during the second and third trimester of pregnancy without any adverse effect on the baby. As women were treated only once, repeated treatments may have resulted in a different outcome. It is reassuring that repeated administration of DHAPQ as IPTp did not increase the occurrence of adverse birth outcomes, indicating that at least this ACT could be safely administered 2–3 times during the second and third trimester of pregnancy [27]. Although the number of women treated and infants followed up is substantial, smaller safety differences between artemisinin-based combinations cannot be excluded. Country-wide post-marketing surveillance would be very helpful in confirming that any of the 4 combinations tested can be safely used to treat malaria during pregnancy.

## Abbreviations

AE: adverse events
AL: artemether-lumefantrine
ACT: artemisinin-based combination treatment
DHAPQ: dihydroartemisinin–piperaquine
Hb: Haemoglobin
IRR: incidence rate ratio
IQR: interquartile range
ITN: insecticide treated net
LBW: low birth weight
LTFU: lost to follow-up
MQAS: mefloquine-artesunate
PRR: pooled risk ratio
PCR: polymerase chain reaction
SAE: serious adverse event
